# The Important Role of p21-Activated Kinases in Pancreatic Exocrine Function

**DOI:** 10.3390/biology14020113

**Published:** 2025-01-22

**Authors:** Irene Ramos-Alvarez, Robert T. Jensen

**Affiliations:** Digestive Diseases Branch, National Institute of Diabetes and Digestive and Kidney Diseases, National Institutes of Health, Bethesda, MD 20812-1804, USA; irene.ramosalvarez@nih.gov

**Keywords:** p21-activated kinases, PAK2, PAK4, CCK-8, pancreas, pancreatic secretion, pancreatic growth, pancreatic cancer, diabetes, insulin secretion

## Abstract

The p21-activated kinases (PAKs) are a conserved family of serine/threonine protein kinases, which are effectors for the Rho family GTPases, namely, Rac/Cdc42. PAKs are divided into two groups: group I (PAK1–3) and group II (PAK4–6). Both groups of PAKs have been well studied in apoptosis, protein synthesis, glucose homeostasis, growth (proliferation and survival) and cytoskeletal regulation, as well as in cell motility, proliferation and cycle control. However, little is known about the role of PAKs in the secretory tissues, including in exocrine tissue, such as the exocrine pancreas (except for islet function and pancreatic cancer growth). Recent studies have provided insights supporting the importance of PAKs in exocrine pancreas. This review summarizes the recent insights into the importance of PAKs in the exocrine pancreas by reviewing their presence and activation; the ability of GI hormones/neurotransmitters/GFs/post-receptor activators to activate them; the kinetics of their activation; the participation of exocrine-tissue PAKs in activating the main growth-signaling cascade; their roles in the stimulation of enzyme secretion; finally, their roles in pancreatitis. These insights suggest that PAKs could be more important in exocrine/secretory tissues than currently appreciated and that their roles should be explored in more detail in the future.

## Simple Summary

The p21-activated kinases are a conserved family of serine/threonine protein kinases, which are well-established effectors for the small GTPases, Rho GTPase Cdc42 and Rac. The p21-activated kinase family is divided into two groups with three members each: group I with PAK1–3; and group II with PAK4–6. PAKs have been extensively studied, particularly in normal tissue development, normal and tumor growth/proliferation and its regulation, cytoskeletal organization/adhesion, cell migration, cell cycle progression, proliferation, aging, immune response, cell survival and CNS function, but their possible role(s) in exocrine secretory tissues has been poorly studied, such as in pancreatic tissues (except for their role in islet function). Numerous recent studies, almost entirely in pancreatic exocrine tissue, show that PAKs play an important role in pancreatic exocrine growth/secretion and disease. This study reviews the limited information from older and new/recent studies that suggest that PAKs might be important not only in pancreatic exocrine tissue function but in other exocrine/secretory organs and, therefore, should be studied in more detail in the future. These recent, summarized results include the presence and activation of PAKs by physiological/pathological stimuli, as well as the signaling cascades involved, which, in a number of cases, are novel.

## 1. Introduction

**Figure 1:**
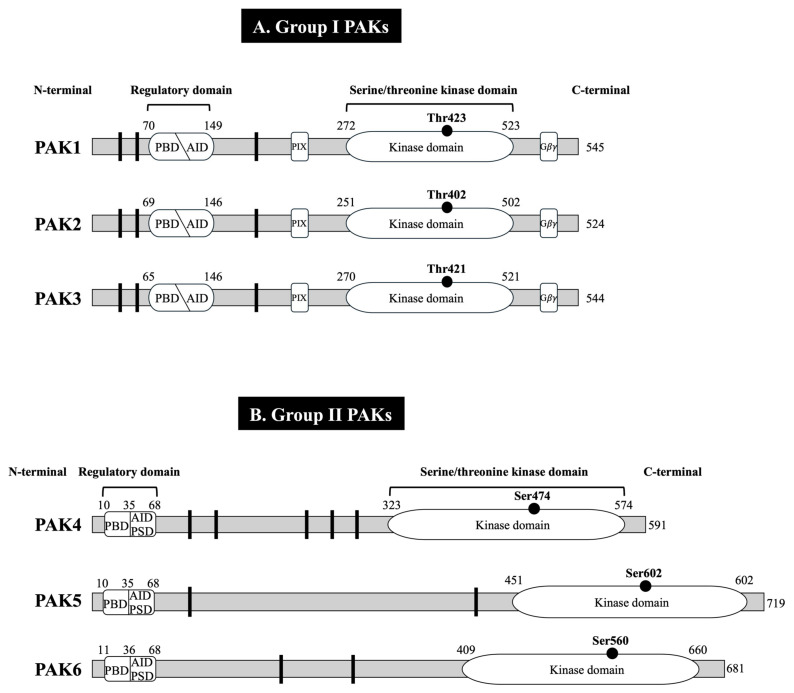
Structure of group I (**A**) and group II (**B**) PAKs. The PAK family consists of six members that are divided into two subgroups according to their sequence homology and structural and activation characteristics: group I (PAK1–3) and group II (PAK4–6) [1,2,3,4,5]. All PAKs have a proline rich region (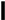), N-terminal regulatory domain and a conserved C-terminal serine/threonine kinase domain. Group I PAKs have a PIX-binding domain, Gβγ-binding domain, and AID behind the PBD, which acts with the PBD as a dimer. Group II PAKs have an additional AID-like domain (PSD) that exists alone [1,3,6,7,8,9]. Numbers indicate the number of the amino acids at the boundaries of various subdivisions. Abbreviations: PBD, p21-binding domain; PIX, PAK-interacting exchange factor; AID, autoinhibitory domain; PSD, pseudo-substrate domain.

The p21-activated kinases (PAKs) are a conserved family of serine/threonine protein kinases, which are well-established effectors for the small GTPases, Rho GTPase Cdc42 and Rac [1,2]. The PAK family consists of six members that are divided into two subgroups according to their sequence homology and structural and activation characteristics: group I (PAK1–3) and group II (PAK4–6) [1,2,3] (Figure 1).

Both groups of PAKs play central roles in many physiological and pathological processes [4,10,11,12,13,14,15,16,17,18,19]. In group I (PAK1–3), the most studied members are PAK1 and PAK2, whose physiological signaling roles have been extensively studied particularly in the regulation of cell survival, apoptosis, cell motility, protein synthesis, glucose homeostasis, growth and cellular proliferation [2,4,10,11,12,16,17,18,19]. Group II PAK’s, with PAK4 being the most studied member, physiological role has been extensively reported in the regulation of cell morphology, cytoskeletal organization, cell proliferation, cell cycle control, migration and growth [6,13,14,15,20,21]. Both group I and II PAKs have also been extensively studied for their roles in numerous pathologic processes, particularly in human cancer (invasion, metastases, apoptosis, epithelial mesenchymal transformation, DNA repair, angiogenesis and drug resistance) and in inflammation, neurological disorders, diabetes, CNS disorders, cardiac disease infectious diseases and cardiovascular diseases [2,7,22,23,24,25,26].

Although both groups of PAKs have been extensively studied for their physiological/pathological roles in the pancreas, there is very little information on their specific roles in pancreatic exocrine function (secretion and growth). This lack of information also extends, in general, to exocrine secretion from other glands (except for insulin) and secretion, in general, of hormones, enzymes, etc., from other glands. A number of recent studies have provided support for the conclusion that both group I and II PAKs might play important essential roles in both pancreatic physiological responses, including pancreatic enzyme secretion and normal pancreatic growth, as well as pathological disorders of the exocrine pancreas, which would likely have important relevance to their possible role(s) in other exocrine/secretory organs. In this paper, we review these recent studies, providing evidence for the importance of PAKs in pancreatic exocrine tissue including in both secretion/growth and the exocrine pancreatic pathological disorder, pancreatitis, as well as the signaling cascades involved, which have a number of unique features not generally reported in the involvement of PAKs in other well-studied tissues.

Prior to dealing specifically with this area, to understand better the various properties of the two PAK families, we first will briefly discuss the structure and activation of these two groups, as well as their pharmacology and signaling from results available from other tissues in which the PAKs have been well studied.

## 2. General Structure and Activation of Group I and II PAKs

Group I and II PAKs share a similar structure: an amino-terminal p21-binding domain (PBD), which can bind Rho GTPases (Cdc42/Rac), and a highly conserved serine/threonine kinase domain at the carboxy-terminal [1,2,8,27,28] (Figure 1). Both PAK groups contain an autoinhibitory domain (AID) after the p21-binding domain [1,2,8,27,28] (Figure 1). Moreover, there is considerable homology within the p21-binding domains between group I and II PAKs [27]. Despite these similarities, both group I and II PAKs have important differences in their structure. Group I PAKs have two proline-rich regions (PXXP) in front of the p21-binding domain; a PAK-interacting exchange factor (PIX)-binding domain that binds PAK-interacting exchange factor between the autoinhibitory domain and the serine/threonine kinase domain; a Gβγ-binding domain behind the kinase domain [1,2,8,27,28] (Figure 1A). In group I PAKs, the autoinhibitory domain overlaps with the p21-binding domain, and together they act as a dimer. Group I PAKs’ activation occurs when active Cdc42 or Rac binds to the p21-binding domain, disrupting the interaction between the autoinhibitory domain and the p21-binding domain, leading to a conformational change of group I PAKs resulting in it becoming a monomer, which subsequently becomes auto-phosphorylated [on Thr423 (for PAK1)] [1,2,8,10,27,28] (Figure 1A and Table 1).

In contrast, the autoinhibitory domain of group II PAKs does not overlap with the p21-binding domain, and it is located behind the p21-binding domain [1,2,8,27,28]. Some older activation models suggest that the autoinhibitory domain of group II PAKs, which keeps them inactive, is an autoinhibitory pseudo-substrate domain (PSD) [1,2,8,27,28] (Figure 1B and Table 1). In group II PAKs, the phosphorylation of the activation loop does not trigger kinase activation but may depend on conformational changes [31,32]. This is contrary to group I PAKs, and most kinases, in which phosphorylation of the activation loop is involved directly in generating kinase activation [8,31]. At present, two mechanisms of the activation of group II kinases, particularly for PAK4, have been proposed [8,27,31,32]. In one model, group II PAKs are activated when active Cdc42 binds to the p21-binding domain and causes a conformational change; thus, PAK4 exists as a monomer in the inactive state and remains inactive due to the binding of the kinase domain and the autoinhibitory domain-like sequence [8,27,31,32] (Figure 1B and Table 1). In the second model, the activation of PAK4 is dependent on the reduction in pseudo-substrate domain auto-inhibition mediated by SH3 proteins; Cdc42 binds to the p21-binding domain, reorienting it, and allows the pseudo-substrate domain to bind to SH3 proteins, resulting in the reduction in auto-inhibition and kinase activation [20,29] (Figure 1B and Table 1). The interaction of the pseudo-substrate domain with the p21-binding domain can keeps PAK4 inactive [29]. Differences and similarities in the structure and activation of group I and II PAKs are explained in more detail in Figure 1 and Table 1.

In numerous studies, the activation of group I PAKs is primarily assessed by the determination of the phosphorylation of threonine in the kinase domain (Thr423 in PAK1, Thr402 in PAK2 and Thr421 in PAK3) (Figure 1 and Table 1) [2,8,9,27,28,30]. In contrast, to assess the activation of group II PAKs, serine phosphorylation in the kinase domain is generally used (Ser474 in PAK4, Ser602 in PAK5 and Ser560 in PAK6) (Figure 1 and Table 1) [2,8,9,27,28,30]. Recently, the assessment of the possible roles of the PAKs in various physiological/pathological processes has been greatly helped by the use of various selective antagonists (Table 2).

For group I PAKs, two inhibitors have been most widely used. These included IPA-3, an allosteric inhibitor with >100-fold greater potency for PAK1 than group II PAKs [2,33,34,42,43], and FRAX597, an ATP competitive antagonist with >10,000 nM for PAK2 than PAK4, respectively [34,42,44] (Table 2). Two different inhibitors have been most widely used for PAK4: the ATP competitive antagonist, PF-3785309, which has a selectivity for PAK4, inhibiting it with 40-fold greater potency than PAK2 [2,35,45,46], and another ATP competitive antagonist compound, LCH-7749944 [2,9,36,47] (Table 2).

## 3. Why Recent Studies of PAK’s Action in Pancreatic Exocrine Function Were Performed?

Isolated results from a number of studies in the literature, performed prior to the recent studies in pancreatic acinar cells, which are reviewed below, provided evidence supporting the possibility that PAKs could be important in the secretion/growth of exocrine pancreas and other exocrine/secretory glands besides its well-studied effects in islets. The primarily function of the exocrine pancreas involves the synthesis and secretion of digestive enzymes/fluid essential for digestion in contrast to the endocrine pancreas, which is involved in the secretion of various hormones, especially insulin, which are essential for many physiological processes. As described briefly above, in general, the effects of PAKs on secretion had not been a well-studied area in any exocrine secretory organ, including the exocrine pancreas. This is in contrast to the endocrine pancreas, where the role of PAKs has been well studied, particularly in response to the insulin synthesis/secretion [6,8,9,11,22,27,28,48,49,50,51,52,53] (Section 6). Nevertheless, some isolated study results are available to suggest that PAKs may play an important role in exocrine gland secretion as well as in exocytosis/secretion by other tissues [1,2,54]. It has been reported that PAKs are involved in mast cell secretion [55,56,57,58,59], and, in the hippocampus, the disruption of PAK1 suppresses inhibitory neurotransmission through an increase in the tonic secretion of endocannabinoids [60]. Furthermore, PAK1 and PAK2 regulate the activation and secretion of TACE/ADAM10 proteases in human embryonic kidney 293T cells [61], and PAK2 is required for platelet secretion [62,63,64]. PAK2 has been found in pituitary secretory granules where it phosphorylates prolactin [65].

Similarly, although the role of PAK’s in exocrine pancreatic growth has not been studied, their roles in tumor growth/aggressiveness/tumor drug resistance have been extensively studied, particularly in pancreatic cancer (Section 8).

## 4. Recent Insights of Group I and II PAKs’ Roles in Exocrine Pancreas: Presence and Activation

### 4.1. Presence of Group I and II PAKs in Exocrine Pancreas

In recent studies using isolated dispersed rat pancreatic acini [10,14], the only group I PAKs found was PAK2 [10], and, similarly, from the group II family, only one member was found, PAK4 [14]. In contrast to these studies in rat pancreatic acini, little is known of the expression of group I or II PAK in pancreatic acinar cells in other species or to compare these results to other studies in rodents. Because of that, we compare the results in rat acinar tissue for similarities and differences from previous studies of PAKs in any pancreatic tissue in rat, mice and human, not only from the few studies in exocrine tissue but also with its expression in pancreatic islets from these species and in pancreatic cancer. Contrary to the rat pancreas results above, PAK1 has been reported by some in mouse acinar tissue [66,67] but not by others [68,69]. Furthermore, PAK3 is reported present in mouse acinar cells [70], whereas none was seen in our studies [10,14]. Similarly, our results differ from findings in human pancreatic acinar tissue where no PAK4 expression was reported in one study [68], whereas in a second study, PAK1 was found [71].

These recent results in rat pancreatic acinar tissue are similar to those reporting the presence of PAK2 in murine pancreatic ß cells [72] and in PAK4 in mouse and human islets [49], as well as in the rat insulinoma cell line INS-1 823/13 [49]. However, they differ from numerous studies that report the presence of PAK1 in mouse islets [67,73,74], as well as in human islets [70,71,74] and in the rat insulinoma cell line INS-1 823/13 [73] and murine ß cell lines, MIN6 cells [70]. Furthermore, they differ from the finding of PAK3 in mouse islets [67,70]. These recent results of finding only these two PAKs present in rat pancreatic acinar cells also differ from the results of PAKs found in pancreatic cancer. In pancreatic cancer, all three group I PAKs (PAK1–3), as well as each of group II PAKs (PAK4–5), except for PAK6, have been reported [59,71,75,76,77,78]. PAK1 and PAK4 have received the most attention because they are most frequently overexpressed [75,76]; however, each of the five PAKs has been shown to be important in pancreatic cancer growth. These results demonstrate that the expression of PAK subtypes differs markedly not only between the same pancreatic tissue in different species but also between the different pancreatic tissues (i.e., exocrine, islets and pancreatic cancer).

### 4.2. Activation of Group I and II PAKs in Exocrine Pancreas

**Figure 2:**
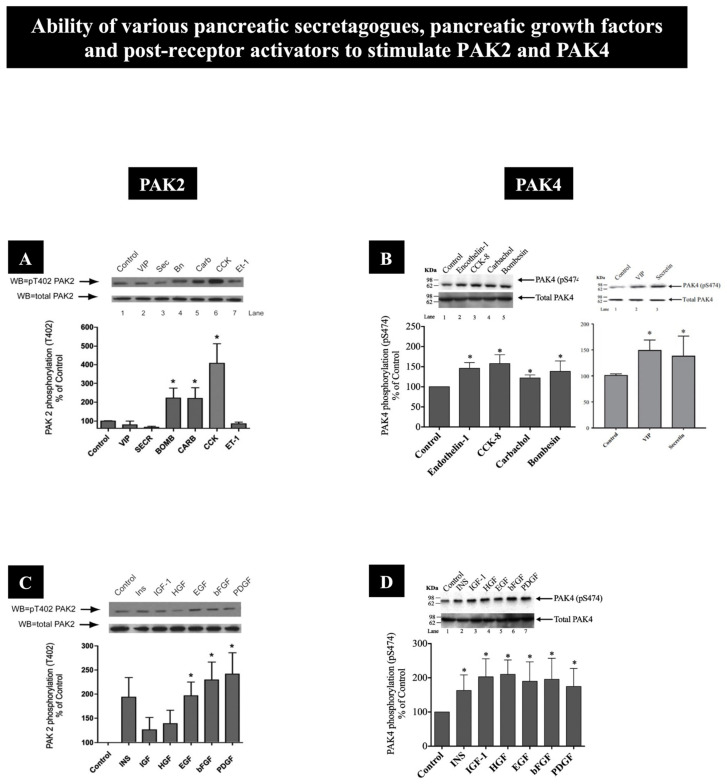
Ability of various pancreatic secretagogues, pancreatic growth factors and post-receptor activators to stimulate activation of PAK2 (**A**,**C**) and PAK4 (**B**,**D**) (i.e., measured by detecting phosphorylation of T402 and S474, which has been shown to be essential for PAK2 and PAK4 protein kinase activity, respectively [9,29,85,86,87,88]) in rat pancreatic acini. (**A**,**B**) Ability of various pancreatic acinar secretagogues [10,14,15,79,83] [cholecystokinin (CCK-8, 1 nM, 1 min), carbachol (10 uM, 1 min), bombesin (1 nM, 1 min), vasoactive intestinal peptide (VIP) (10 nM, 1 min), secretin (10 nM, 1 min) or endothelin-1 (10 nM, 1 min)] to activate PAK2 or PAK4 in isolated pancreatic acini. (**C**,**D**) Ability of various pancreatic growth factors [10,14,15,79,83] [insulin (INS, 1 uM, 10 min), insulin-like growth factor 1 (IGF-1, 100 nM, 10 min), hepatocyte growth factor (HGF, 1 nM, 10 min), epidermal growth factor (EGF, 10 nM, 5 min), basic fibroblast growth factor (bFGF, 100 ng/mL, 5 min) and platelet-derived growth factor (PDGF, 100 ng/mL, 10 min)] to activate PAK2 or PAK4 in the pancreatic acini. Isolated pancreatic acini were incubated in the absence or presence of these pancreatic secretagogues, pancreatic growth factors and post-receptor activators at the indicated concentrations and time incubations and then lysed. The cell lysates were subjected to Western blotting and analyzed using anti-pT402 PAK2 or anti-pS474 PAK4 and, as loading control, anti-total PAK2 or PAK4. Bands were visualized using chemiluminescence and quantified by densitometry. Results are expressed as % control phosphorylation. Top: Results of a representative blot of five independent experiments. Bottom: Mean ± SE of six independent experiments. *, *p* < 0.05 compared with the control. These results are modified from the figures and data in [10,14,15]. These results show that various pancreatic growth factors and secretagogues can activate both PAK2 and PAK4 in pancreatic exocrine tissue, but they are similar with some stimulants and differ with others.

**Figure 3:**
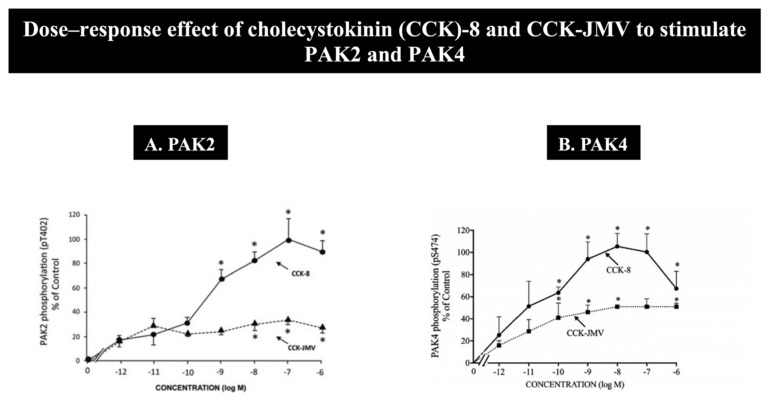
Dose–response effect of cholecystokinin (CCK)-8 and CCK-JMV (a synthetic CCK analog that distinguishes high/low affinity CCK_1_-R receptor states [10,14,89,90,91]) to activate PAK2 (**A**) and PAK4 (**B**) (i.e., measured by detecting phosphorylation of T402 and S474, which has been shown to be essential for PAK2 and PAK4 protein kinase activity, respectively [9,29,85,86,87,88], in rat pancreatic acini. Isolated pancreatic acini were incubated in the absence or presence of CCK-8 and CCK-JMV (at the indicated concentrations) for 3 min and then lysed. Results are expressed as means ± SE of three independent experiments. Results are expressed as % of basal stimulation of the control group (PAK2: CCK-8 100 nM; 904 ± 140% of control. PAK4: CCK-8 1 nM; 193 ± 15% of control). * *p* < 0.05 compared with the control. These results are modified from the figures and data in [10,14]. These results show that both activation of the high and the low affinity CCK_1_-R receptor states are need for full activation of either PAK2 or PAK4 by CCK.

Recent studies [10,14,15] report that both PAK2 and PAK4 present in rat pancreatic acinar tissue are activated by numerous pancreatic physiological secretagogues, including cholecystokinin (CCK), muscarinic cholinergic agonists (carbachol) and bombesin/GRP, which all activate the phospholipase C (PLC) signaling cascade [79,80] (Figure 2A,B and Table 2). However, only PAK4 [10,14,15] was also activated by the hormone/neurotransmitter, endothelin-1, which activates neither PLC nor the cyclic AMP signaling cascade in pancreatic acinar cells [81], as well as was activated by secretin and VIP, which increase cAMP-stimulated signaling cascades [15,82,83,84] (Figure 2B and Figure 3B and Table 2). PAK2 and PAK4 also are activated by numerous pancreatic growth factors [10,14]. While the growth factors EGF, bFGF and PDGF stimulated both PAK2 and PAK4 in pancreatic exocrine tissue, insulin, IGF-1 and HGF only had an effect on PAK4 activation [10,14] (Figure 2C,D and Table 2). Moreover, the stimulation of both pancreatic acinar cell PAK2 and PAK4 by post-receptor activators was only possible with the phorbol ester, TPA, which activates PKC (Table 2), whereas agents stimulating changes in [Ca^2+^]_i_, such as thapsigargin, the Ca^2+^ ionophore, and A23187 (Table 2) or the post-receptor activators of cAMP, 8-Br-cAMP and forskolin (Figure 3A) only stimulated PAK4 [10,14]. These differences, in which pancreatic secretagogues and growth factors and post-receptor activators stimulated PAK2 and/or PAK4, demonstrate that the cellular activation signaling pathways vary markedly between group I (PAK2) and group II (PAK4) PAKs in pancreatic exocrine tissue (Section 5) (Figure 2A–D and Figure 3A and Table 2).

A comparison of these results of the ability of these receptor and post receptor stimulants to activate group I and groups 2 in pancreatic exocrine tissue show both similarities and marked differences in some respects from that reported by these stimulants in other tissues (Table 3).

Similar to the findings in pancreatic acinar cells [10], PDGF and FGF stimulated the activation of group I PAKs, whereas insulin did not, in NIH-3T3 cells [99]; EGF activated PAK2 in mouse skin epidermal cells [103]; EGF and carbachol stimulated a group I PAK in Cos7 cells [92]; and bFGF stimulated cell growth by activating PAK1/PAK2 in PC-12 cells [104,105,106]. Similar to the pancreatic acinar cell findings, group I PAKs are activated in a PLC-dependent manner by angiotensin II [107,108] in vascular smooth muscle cells [107,108], gastrin in colorectal cancer cells and colorectal mucosa cells [109,110] or by Rac1, which is a PAK activator in other tissues as well as group I PAK activation by muscarinic cholinergic agents in fibroblasts or neuroblastoma cells [92] and in smooth muscle cells [111]. Different from what is described above in pancreatic acini with PAK2 [10], endothelin did not activate group I PAKs in myocytes [93]; group I PAKs mediated IGF-1 and insulin signaling in mesothelial cells [115] and in mouse endocrine L cells [116], respectively; HGF regulated PAK1/PAK2 in prostate cancer [117] and epithelial cells [112,118]; and PKA activation was required for PAK activation by other GPCRs [94]. As described in pancreatic acinar cells with PAK4 [14], IGF-1 or PDGF can active PAK4 [9,29]; HGF, EGF and insulin activates PAK4 in epithelial cells [100,101,102]. Although there are no studies on the ability of VIP or secretin to activate PAK4, some studies reported that the activation of some G-protein-coupled receptors, such as those for ß-adrenergic agents, prostaglandins and alpha-MSH, can stimulate PAK4 activation via cAMP in HEK293 cells (human embryonic kidney 293 cells), B16 melanoma cells and MCF7 (breast cancer cells) [94,95,96]. Similarly, it is reported in different cancers with agents that activate cAMP that they can activate PAK4, such as thyroid-stimulating hormones in papillary thyroid cancer [97], C-X-C motif chemokine 12 in prostate cancer [119] and alpha-MSH in B16 melanoma cells [96].

Previous studies have demonstrated that PAK2 and PAK4 can be activated by post-receptor activators in other tissues, as described in the pancreas [10,14,15]. The post-receptor activators of the cAMP pathway, 8-Br-cAMP and forskolin activated Cdc42 in human mesangial cells [113], which is the principal upstream activator of PAK4 [1,2,6,8,27,28]. Moreover, forskolin can activate PAK4 in papillary thyroid cells [97] and in prostate cancer cells [114].

### 4.3. Dose–Response Effect on Group I and II PAK Activation in Exocrine Pancreas

The hormone/neurotransmitter, CCK, is one of the most important physiological regulators of pancreatic exocrine functions (i.e., the secretion, growth and synthesis of enzymes) [80,120,121,122], as well as is important in various exocrine pancreatic pathophysiological processes (such as pancreatitis and cancer growth) [80,120,123,124,125,126]. Numerous studies demonstrate that CCK mediates its actions in various tissues by interacting with the G-protein coupled receptors CCK_1_-R or CCK_2_-R, with CCK_1_-R being specific for CCK, and mediating CCK’s action in the pancreas [80,83,127,128,129,130,131,132,133]. CCK can activate both a high- and low-affinity CCK_1_ receptor state, which can mediate different cellular responses [80,89,128]. In pancreatic acinar cells, by using the synthetic CCK analogue, CCK-8-JMV, which is a full agonist for the high-affinity CCK_1_ receptor state and an antagonist for the low-affinity state in rat pancreatic acini [89,90], it has been found in pancreatic acinar cells that PAK2, as well as PAK4 activation, requires the activation of both high and low CCK_1_-R states [10,14] (Figure 3 and Table 2).

For maximal PAK2 activation, both receptor states are required, with 26% of the full activation of the high-affinity CCK_1_ receptor state and 74% of the activation of the low-affinity CCK_1_ receptor state [10] (Figure 3A and Table 2), while 60% of PAK4 maximal CCK-stimulation is due to the activation of the high-affinity CCK_1_ receptor state and 40% to the activation of the low-affinity CCK_1_ receptor state [14] (Figure 3B and Table 2). These studies established that the degree of participation of the high or low affinity state activation that is required for the maximal stimulation of PAK2 and PAK4 varies despite the fact that they are closely structurally related. As recently described with PAK2 and PAK4 in pancreatic exocrine cells [10,14], the activation of both high- and low-affinity CCK_1_ receptor states, although with differences in their relative importance, is also required for the CCK-induced activation of the Src kinases (Lyn and Yes) [134,135], the focal adhesion kinases (p125FAK and PYK2) [128,136], paxillin [128,137] and PKD [91] in pancreatic acini [134]. In contrast to PAK2 and PAK4 in pancreatic acinar cells, CCK-mediated activation in the pancreatic acini of phospholipase D or PI3K requires the activation of only the high-affinity receptor state [138], whereas in the case of PKC-δ [139], CRK-II [140] or SFK activity [141], only the low-affinity CCK_1_ receptor state is required for activation.

**Figure 4:**
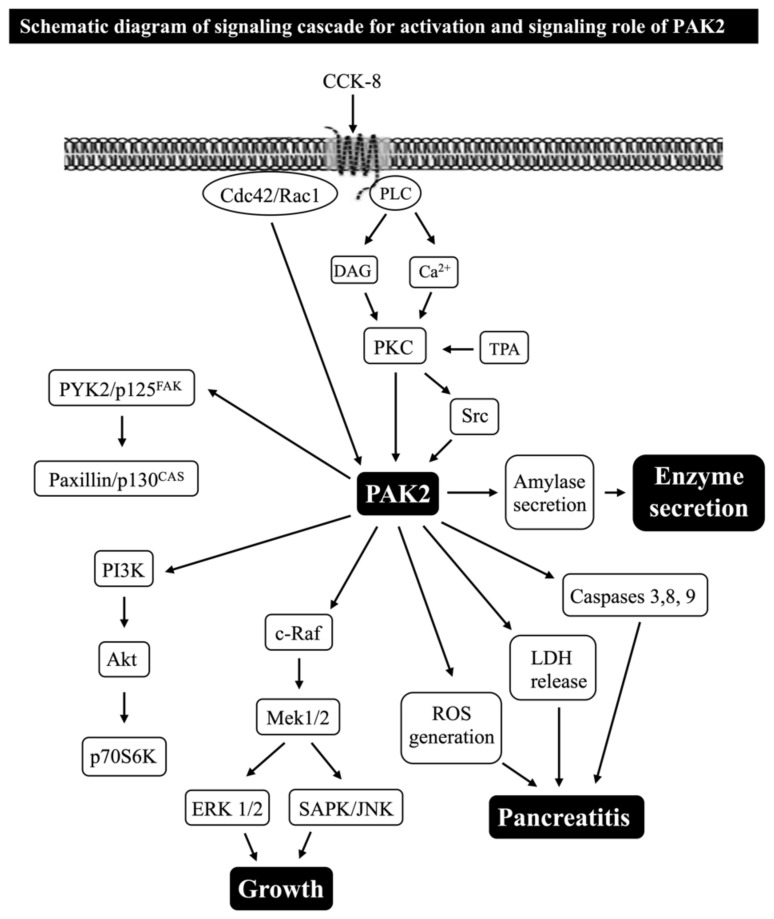
Schematic diagram summarizing the signaling cascade for CCK activation and signaling roles of PAK2 from recent studies in rat pancreatic acinar cells [10,12]. In rat pancreatic acinar cells, maximal activation of PAK2 (i.e., measured by detecting phosphorylation of T402, which has been shown to be essential for PAK2 protein kinase activity [85,86,87,88]) requires activation of Cdc42/Rac1 and phospholipase C, resulting in PKC activation. PAK2 activation involves both PLC-dependent and independent cascades with the PLC-dependent cascade mediated by PKC activation, with changes in Ca^2+^ not being involved. PAK2 also requires SFK-dependent and SFK-independent signaling. However, changes in PI3K are not involved, but a major component of PAK2 activation mediated by CCK-activation is Cdc42/Rac1 [10]. Activation of PAK2 is needed to stimulate several signaling kinases including MAPKs (Mek1/2, p44/42 and JNK); FAKs (p125^FAK^ and PYK2); scaffolding proteins (paxillin and p130^CAS^); caspases 3, 8 and 9; LDH release; and ROS generation, which are important in mediating numerous cellular functions [10,12]. The activation of PAK2 has a unique dual role in altering the activity of the PI3K–Akt pathway, which is required for the stimulation of p85, and also in mediating the inhibition of Akt activity via the dephosphorylation of Akt [10,12]. These results are drawn from the figures and data in [10,12]. These results show that PAK2 activation is essential for the CCK-mediated activation of pancreatic acinar growth cascades and enzyme secretion, as well as in CCK-mediated experimental pancreatitis. However, PAK2 was not activated by VIP or secretin, the main stimulants for fluid and electrolyte secretion.

**Figure 5:**
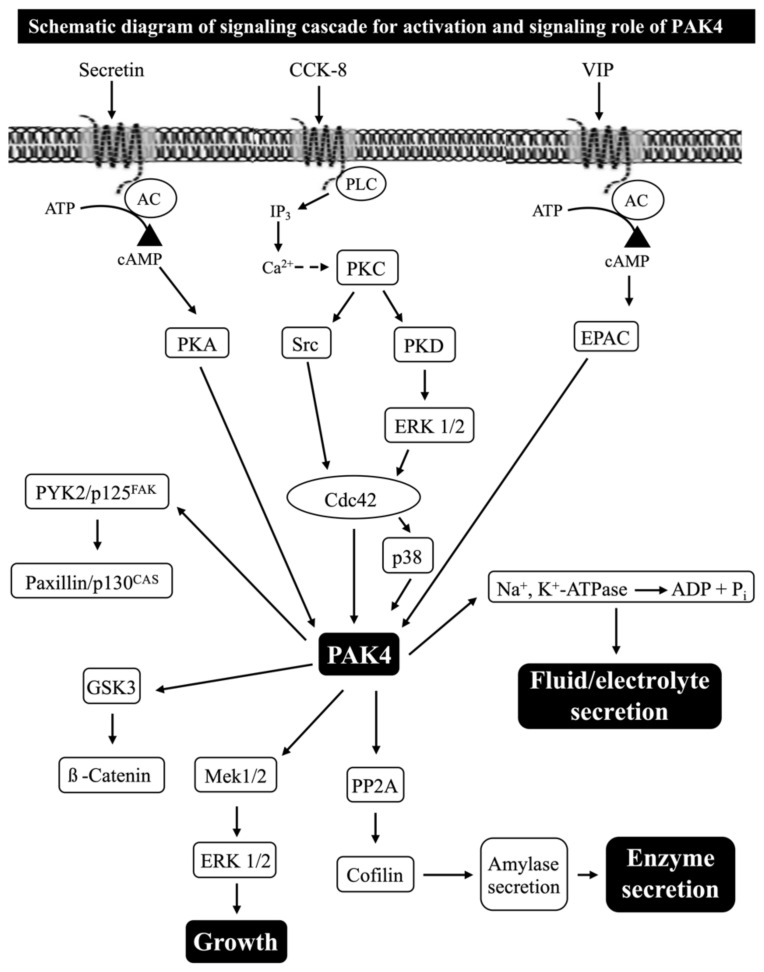
Schematic diagram summarizing the signaling cascade for activation and signaling role of PAK4 in response to CCK, secretin or VIP from recent studies [14,41,143] in rat pancreatic acinar cells. Maximal activation of PAK4 by CCK-8 (i.e., measured by detecting phosphorylation of S474, which has been shown to be essential for PAK4 protein kinase activity, as well as reflecting its degree of activation, Refs. [9,29]), requires activation of primarily PKC, with a lesser contribution by changes in cytosolic calcium. PKC mediates both Src family kinase (SFK) and protein kinase D (PKD) activation with the latter resulting in ERK1/2 activation and Cdc42 activation. This in turn can stimulate PAK4 activation partially via a p38 mechanism [14]. However, changes in PI3K are not involved in pancreatic acini in contrast to what is reported in a number of other tissues with other stimulants [45,144]. The ERK1/2 and PAK4 inhibition studies demonstrate that CCK-8-mediated ERK1/2 activation and PAK4 activation reciprocally regulate each other’s activation [14]. Activation of PAK4 by secretin in pancreatic acinar cells requires activation of PKA and by VIP requires EPAC; however, both secretin and VIP induced CREB phosphorylation through EPAC. PAK4 activation is important for Na^+^, K^+^-ATPase phosphorylation, and Na^+^, K^+^-ATPase activity [15]. Activation of PAK4 is needed to stimulate several signaling kinases including MAPKs (Mek1/2 and p44/42), FAKs (p125^FAK^ and PYK2); scaffolding proteins (paxillin and p130^CAS^); GSK3 and ß-Catenin; and PP2A and cofilin, which are important in mediating numerous cellular functions [14,40,41]. These results are drawn from the figures and data in [14,15,40,41]. These results show that the signaling cascades for secretin, CCK and VIP mediated the PAK4 activation and stimulation of acinar growth, enzyme secretion and fluid/electrolyte secretion. It is unknown what role, if any, PAK4 plays in CCK-induced experimental pancreatitis.

In pancreatic acinar cells, as in a number of other tissues [79,80,82,142], one of the principal signaling cascades with activation of CCK_1_-R is the stimulation of phospholipase C (PLC), resulting in the generation of inositol phosphates and diacylglycerol, which in turn results in the mobilization of the cellular calcium and activation of PKCs, respectively [79,80,128] (Figure 4 and Figure 5).

A recent study in pancreatic acinar cells found that the activation of PKC, but not changes in cytosolic calcium, by CCK_1_-R stimulation, is an important mediator for the activation of PAK2 and that 40% of the PKC-independent activation of PAK2 was also PLC-independent [10] (Figure 4). In addition to PKC being involved as an upstream activator of the CCK- and TPA-induced activation of PAK2, this same study suggested that the activation of the high-affinity CCK receptor state requires the activation of the Src-family of kinases (SFKs) for full PAK2 activation [10] (Figure 4). In regard to PAK4 activation in pancreatic acinar cells, another recent study reported that CCK_1_-R mediates PAK4 activation primarily through PKC activation and, to a lesser extent, through Ca^2+^ mobilization activation and through both PKD-dependent and independent, as well as SFK signaling cascades [14] (Figure 5). These results demonstrate that CCK_1_-R activation of PAK2 and PAK4 in the same cell can have both marked similarities and differences.

## 5. Recent Insights into the Downstream Signaling Cascades of p21-Activated Kinases in Pancreatic Exocrine/Acinar Tissue (Downstream)

Until recent studies, there was no information on how the activation of group I and II PAKs in pancreatic acinar tissue interfaces with the main cell signaling pathways (i.e., c-Raf/MEK/ERK [80,82,145]; the PI3K/Akt/mTor pathway [80,82,146,147,148,149]; and GSK3 and ß-catenin pathways [79,90,101,139,150,151,152]).

In recent studies in rat pancreatic acinar cells, the CCK_1_-R activation of both PAK2 and PAK4 has been shown to activate the principal growth/proliferation cascade [121,153], which requires MAPK activation, through Mek1/2 and p44/42 [10,12,14,41] (Figure 4 and Figure 5 and Table 2). In addition, these recent studies show that both PAK2 and PAK4 are important for the CCK_1_-R activation of the focal adhesion pathways involving PYK2/p125^FAK^ and for the activation of the important scaffolding/adapter proteins, paxillin/p130^CAS^ [12,41] (Figure 4 and Figure 5 and Table 2). Despite these similarities of the roles of PAK2 and PAK4 in the above signaling cascades, there were major differences in the ability of PAK2 or PAK4 to stimulate the PI3K/Akt pathway, which is an important regulator of apoptosis and cell cycle progression in the pancreas [80,122,146,147,148,154]. While PAK2 is an important mediator of the CCK-induced phosphorylation of p85 PI3K and the ability of CCK-and TPA to alter the activation of the PI3K pathway by stimulating dephosphorylation of Akt and p70S6K [12] (Figure 4 and Table 2), PAK4 did not regulate p85 PI3K or Akt nor p70S6K activation [41] (Figure 5 and Table 2). This latter finding differs from the findings in gastric cancer [45], wherein PAK4 is an important regulator of the activation of the PI3K pathway. Another difference between the activated downstream signaling cascades of group I (PAK2) and group II PAKs (PAK4) in pancreatic acinar tissue is that CCK, TPA, secretin and VIP stimulated the activation of ß-catenin and its upstream mediator GSK3 in a PAK4-dependent manner; however, this was not dependent on PAK2 [12,41] (Figure 4 and Figure 5 and Table 2). The latter result contrasts with the ability of the CCK_2_-R agonist, gastrin, to activate β-catenin signaling, which was mediated by a PAK1-dependent pathway in gastric epithelial cells [37]. A recent study [40] in rat pancreatic acini demonstrated that PAK4 activation is essential in the CCK-mediated activation of cofilin, which is essential for mediating CCK-stimulated growth and enzyme secretion; however, at present, it is unknown if the activation of group I PAK, PAK2, in these cells is also involved [40] (Figure 5 and Table 2).

The recent studies in pancreatic acinar cells reporting that the activation of both group I (PAK2) and group II (PAK4) PAKs [12,41] can result in stimulation or interaction with various adapter/scaffolding proteins, such as p130^cas^ and paxillin, are similar to reports previously in a number of studies in other tissues in the literature. A number of studies report that the activation of various group I PAKs (PAK1 and PAK3) results in the interaction/activating of the α isoforms of paxillin in different cell lines (including the fibroblast cell line, 3Y1, and NRB and CHO.K1 cells) [155,156,157]; the activated PAK1 down-regulated p130^CAS^ in airway smooth muscle cells [158], and PAK4 activation is needed for stimulation for the activation of paxillin in lung cancer cells [159], as well as studies reporting that PAK4 mediates the activation of paxillin in PAK4-transfected MDCK cells [144] and in DU145 prostate cancer cells [160].

Despite the limited information on the ability of PAK4 to regulate the activity of the focal adhesion kinases (PYK2 and p125FAK) or the adapter protein, p130^CAS^ in the pancreas, PAK4 stimulation is needed for the activation of 125^FAK^ in A549 human lung cancer cells [159], and PAK4 activation is associated with morphologic cellular changes in focal adhesions in NIH 3T3 and in prostate cancer [160,161]. However, in valvular interstitial cells with periostin/B1 integrin activation, PAK1 activation is downstream to p125^FAK^ activation [162].

In recent studies [12,41], the CCK_1_-R activation of the MEK/ERK pathway was dependent on the activation of both group I (PAK2) and group II (PAK4) PAKs by GTPases [12,41]. However, neither PAK2 or PAK4 was required for the activation of the p38 signaling cascade, and the roles of these two different PAKs differed in the activation of the JNK signaling cascade or the activation of c-Raf, with PAK2 activation required but not PAK4 activation [12,41]. Similarly, it has been described in the literature with different members of the MAPK cascade that CCK activates Mek/ERK by a Raf-dependent mechanism [145] and that GTPases are involved in the upstream signaling for JNK activation [163]. Moreover, the group I PAK, PAK1, has been described as a mediator of JNK activation in vascular smooth muscle cells [108]. Furthermore, in numerous normal and tumor cells with various stimuli [164,165], PAK4 activation mediates ERK1/2 stimulation, including in pancreatic cancer cells, in gastric cancer cells and in A549 human lung cancer cells [36,45,68,159], as well as stimulating the Mek1/2 cascade in gastric cancer cells and other tissues [45,164]. However, despite PAK4 leading to Mek/ERK activation in pancreatic acinar cells [41], this is not the case in colon cancer cells where the Raf/Mek/ERK pathway is independent of PAK4 activation [100]. Furthermore, in human epithelial cells, PAK4 activation is needed for p38 stimulation, which is required for enhanced MUC5A main transcription [102], and there is a requirement for the PAK4 activation of JNK in a number of tissues [166,167]. Despite the similarity between group I and II PAKs, the role of both groups of PAKs in the stimulation of MAPK pathways in different cells can vary markedly. Furthermore, within the same cell, such as in pancreatic acinar cells, with the same stimulant (i.e., CCK), the role of group I and group II PAKs (i.e., PAK2/PAK4) in the activation of the different MAPKs can differ. Taken together, these studies demonstrate the importance of PAKs in mediating the CCK- and TPA activation of different MAPKs in pancreatic acinar cells and suggest a participatory role of PAKs in many of the physiological and pathophysiological processes controlled by this pathway in pancreatic acini such as proliferation, regeneration, growth, inflammation and apoptosis [121,153,168,169,170].

A previous study performed in pancreatic acini reported that PAK2 was involved in both dual actions of CCK and TPA on PI3K because PAK2 inhibition reversed both dual actions of CCK and TPA on this pathway by reducing their induced activation of p85 PI3K while reversing its inhibitory effect on Akt activation [12] (Figure 4 and Table 2). However, these results are in contrast with the literature where PAK1 inhibition inhibits Akt [33,62] and GSK-3-β activity [37,62,171,172], and they demonstrate that the activation of PAK2 has a variable role on PI3K/Akt activation in various cells and that with CCK in pancreatic acini, it has a number of novel features. Despite the role of PAK2 in PI3K/Akt activation, all of the changes in Akt were transmitted to its downstream effector P70s6k [173,174]. At present, the mechanisms of these inhibitory effects of CCK on the activity of the Akt pathway in pancreatic acinar cells are unclear [146]; additionally, whether PKC, a PAK2 activator [10] and an Akt inactivator, in other studies [175], is involved in the CCK actions on Akt mediated by PAK is still unknown and requires further research. Similar to the recent findings in pancreatic acinar cells with PAK4 [41], PAK4 activation was independent of PI3K/Akt activation in colon cancer cells [100]. However, PAK4 activation is required for Akt-mediated NF-kB activation and maximal stimulation in pancreatic cancer cells [68,176], for Akt-induced chemoresistance in cervical or gastric cancer cells [45,177], for Akt-mediated enhanced proliferation/invasion in breast cancer cells [178] and for a number of other Akt-stimulated changes in other tumors [179,180]. These results demonstrate that PAK2 and PAK4 involvements with the PI3K/Akt signaling cascade shows wide variation in different cells and that it can differ markedly from the activation of one to another PAK.

Previous studies demonstrate that the CCK and gastrin-related peptides can activate the ß-catenin and GSK3 pathways in both normal and tumor cells [181,182], and, in the pancreas, their activation by CCK could lead to pancreatitis and the stimulation of protein synthesis and growth, as well as pancreatic regeneration after injury and pancreatic development, and act as an important mediator of pancreatic secretion [183,184,185,186,187,188]. Similar to recent results in pancreatic acinar cells [41], PAK4 regulates melanogenesis via the ß-catenin/MITF pathway [96]; PAK4 activates α-MSH/UVB-induced melanogenesis via Wnt/ß-catenin [96]; and the inhibition of PAK4 attenuates nuclear ß-catenin, which reduces the migration, invasion and/or growth in A549 human lung cancer cells [159] and colon cancer cells [189], as well as the growth of normal cells [159].

Most of the studies of the role of group I and II PAKs have been performed with chemical inhibitors (Table 2), such as FRAX597 and PF-3758309 (ATP competitive; PAK2 and PAK4 inhibitors, respectively). These inhibitors were the first ones to be described and are the most used to date. Currently, they are used to study the role of PAK2 and PAK4 in other tissues. Some of these studies included comparative findings with siRNA [40], showing no differences between these inhibitors and the findings using other methods (i.e., siRNA), proving the specificity of these inhibitors for each PAK. However, new inhibitors with higher selectivity among PAKs will help to better understand the specific role of each one of these PAKs.

## 6. Secretion (Amylase Release and Fluid/Electrolyte Secretion)

**Figure 6:**
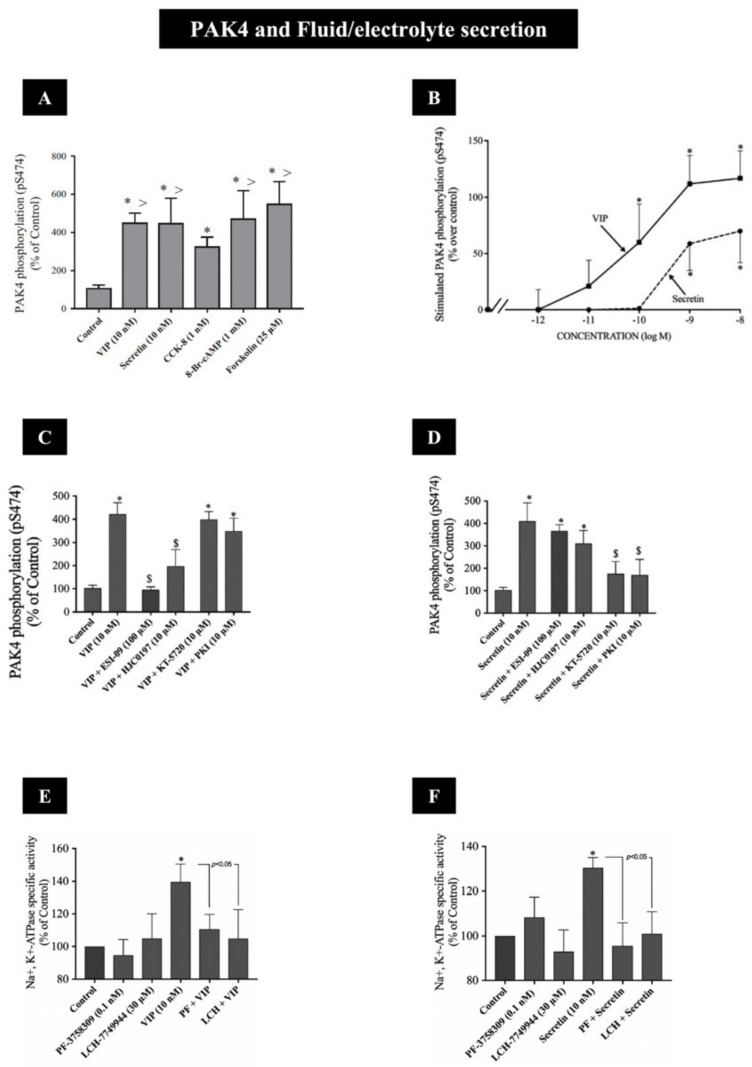
(**A**) Effect of VIP, secretin, CCK-8, 8-Br-cAMP and forskolin on PAK4 activation in rat pancreatic acini. Isolated pancreatic acini were incubated in the absence or presence of VIP (10 nM), secretin (10 nM), 8-Br-cAMP (1 mM) or forskolin (25 uM) for 15 min and CCK-8 (1 nM) for 3 min and then lysed. (**B**) Dose–response effect of VIP and secretin to stimulate PAK4 activation in rat pancreatic acini. Isolated pancreatic acini were incubated in the absence or presence of VIP and secretin (at the indicate concentrations) for 15 min and then lysed. (**C**,**D**) Effect of ESI-09 and HJC0197, EPAC inhibitors, and KT-5720 and PKI, PKA inhibitors, on PAK4 activation by VIP (**C**) and secretin (**D**) in rat pancreatic acini. Isolated pancreatic acini were incubated in the absence or presence of ESI-09 (100 uM), HJC0197 (10 uM, 2h), KT-5720 (10 uM) or PKI (10 uM) for 1 h and then incubated with no addition (control) or secretin (10 nM) for 15 min and then lysed. (**E**,**F**) Effect of PF-3758309 and LCH-7749944, PAK4 inhibitors, on VIP (**E**) and secretin (**F**) stimulation of Na^+^, K^+^-ATPase activity rat pancreatic acini. Isolated pancreatic acini were incubated in the absence or presence of PF-3758309 (0.1 nM) and LCH-7749944 (30 uM) for 3 h and then incubated with no addition (control), VIP (10 nM) or secretin (10 nM) for 15 min and then lysed. PAK4 was measured by detecting phosphorylation of S474, which has been shown to be essential for PAK4 protein kinase activity [9,29]) and Na^+^, K^+^-ATPase activity was measured using a colorimetric assay [190]. Results are expressed as means ± SE of 4 independent experiments. Results are expressed as percentages of basal stimulation of the control group in (**A**,**C**–**F**), and results are expressed as percentages of stimulation over the control group in (**B**) (VIP 10 nM: 217 ± 24% of control). *, *p* < 0.05 compared with the control; >, *p* < 0.05 compared with CCK-8 alone; $, *p* < 0.05 compared with secretin or VIP alone. These results are modified from the figures and data in [15]. These results show that CCK, VIP and secretin can all activate PAK4 in pancreatic exocrine tissue; however, their cellular signaling cascades differ (see Figure 5).

Recent studies show for the first time that the activation of both group I PAKs (PAK2) and group II PAKs (PAK4), by the principal physiological pancreatic stimulant, CCK, is essential for the stimulation of pancreatic enzyme secretion [10,14,40,41] (Figure 4 and Figure 5). Complementary to these studies, recent findings show that the stimulation of both the pancreatic acinar cells PAK2 and PAK4 by post-receptor activators was only possible with the phorbol ester, TPA, which activates PKC (Table 2), whereas agents stimulating changes in [Ca^2+^]_i_, such as thapsigargin, the Ca^2+^ ionophore, A23187 (Table 2) or the post-receptor activators of cAMP, 8-Br-cAMP and forskolin (Figure 6A) only stimulated PAK4 [10,14].

Furthermore, the activation of PAK4, by some of these post-receptor activators, has been shown to be essential for the activation of pancreatic acinar Na^+^, K^+^-ATPase, which mediates fluid and electrolyte secretion from acinar cells, stimulated by the neurotransmitters/hormones, secretin and vasoactive intestinal peptide (VIP) (Figure 2B and Figure 6 and Table 2). The detailed studies [10,14,40] of the signaling cascades involved with enzyme secretion with PAK2 (Figure 4) and PAK4 (Figure 5) activation demonstrate that the primary signaling cascades for both PAKs with CCK result in the activation of phospholipase C. The subsequent stimulation of SFKs, with the activation of Cdc42/Rac1 also participating, as well as PKD and ERK activation with PAK4 (Figure 4 and Figure 5), can be observed. A more recent study [40] demonstrated that an essential signal member for the stimulation of pancreatic enzyme secretion by activating PAK4 was the activation of protein serine phosphatase 2A (PP2A), resulting in the stimulation of cofilin, which played a pivotal convergent role for a number of other signal cascades participating in pancreatic enzyme secretion [40] (Figure 6). In the case of the PAK4 activation of pancreatic acinar Na^+^, K^+^-ATPase, which mediated VIP/secretin-stimulated fluid and electrolyte secretion (Figure 6E,F) [15] in pancreatic acinar cells, the essential signaling pathway activating PAK4 was the stimulation of adenylate cyclase resulting in the activation of both protein kinase A and EPAC (Figure 6C,D) [15]. In contrast to PAK4 (Figure 2A, Figure 5 and Figure 6B and Table 2), VIP/secretin did not activate PAK2 in pancreatic acinar cells and thus was not involved in VIP/secretin-stimulated fluid/electrolyte secretion in these cells (Figure 4 and Table 2) [10].

The recent findings in pancreatic acinar cells showing an essential role for PAKs in pancreatic enzyme secretion, as well as pancreatic acinar fluid and electrolyte secretion, suggest that PAKs may play a much larger role than currently appreciated in secretion/exocytosis from other exocrine glands and secretory tissues including in neural tissue, with the synaptic release of neurotransmitters in both in the CNS and periphery. This conclusion is supported both by the extensive literature reporting the involvement of both group I and group II PAKs in insulin secretion [9,49,50,53] and by isolated studies in the literature that report the involvement of group I PAKs in secretion from a diverse variety of exocrine/secretory cells including from mast cells [57,59,191]; in secretion from pituitary cells [65]; in secretion from platelets [62,63,64]; in the secretion of RACE/ADA10 proteases from both embryonic kidney 293 cells as well as cancer cells [61,192]; in acrosomal exocytosis during sperm capacitation [193]; in the secretion of GLP-1 from intestinal endocrine L cells [194]; in GABA secretion/release during synaptic transmission [195]; in stimulation exocytosis from bovine chromaffin cells [196]; in secretion from vascular smooth muscle cells [197]; and in regulating inhibitory neurotransmission in hippocampal cells [60].

## 7. Acute Pancreatitis

**Figure 7:**
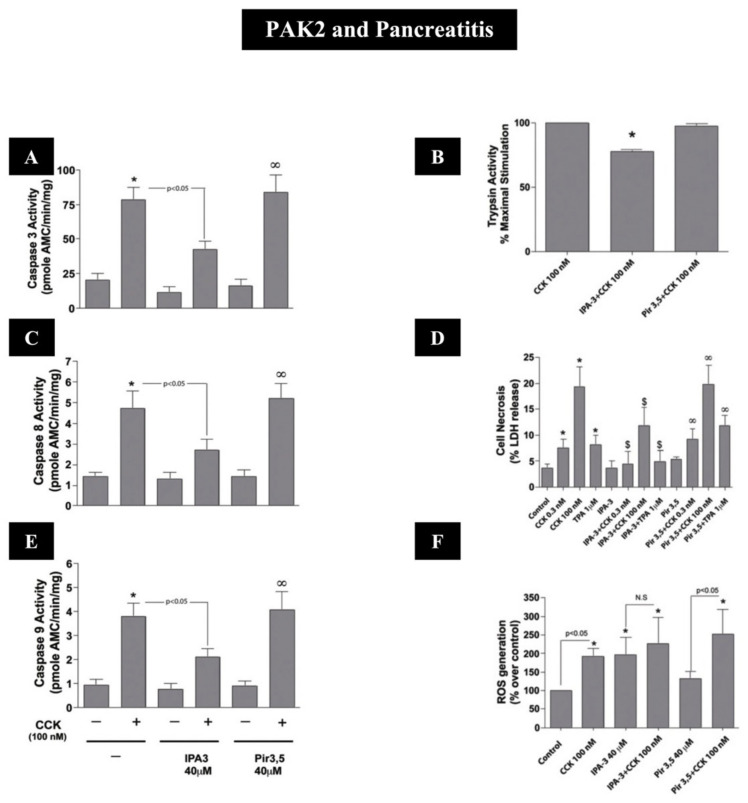
Effect of inhibition of PAK2 on supramaximal CCK-mediated experimental pancreatitis induced apoptosis, trypsin activation and cell necrosis in pancreatic acini. (**A**,**C**,**E**) Freshly isolated rat pancreatic acini were pre-incubated with either 40 uM IPA-3, a specific group I PAK inhibitor [10,12,33,85] or Pir 3,5 (an inactive control) [10,12] for 3 h. Caspase (caspases 3 (**A**), 8 (**C**), 9 (**E**)) activities were measured as previously reported [12]. The results are representative of 4 independent (*n* = 4) experiments. Results shown are the means ± SE. *, *p* < 0.05 compared with the control; ∞, *p* < 0.05 compared with Pir 3,5 alone. (**B**) Freshly isolated rat pancreatic acini were pre-incubated with either 40 uM IPA-3 or Pir 3,5 for 1 h followed by stimulation with 0.3 and 100 nM CCK and 1 uM TPA for 20 min. Trypsin activity was measured as previously reported [203]. The results are representative of 4 independent (*n* = 4) experiments. The data are expressed as the percentage of maximal activity obtained when acini were incubated for 20 min with 100 nM CCK. Results shown are the means ± SE. *, *p* < 0.05 compared with CCK maximal stimulation. (**D**) Freshly isolated rat pancreatic acini were pre-incubated with either 40 uM IPA-3 or Pir 3,5 for 1 h followed by stimulation with 0.3 and 100 nM CCK and 1 uM TPA for 1 h. LDH release was measured as previously reported [203]. The results are representative of 4 independent (*n* = 4) experiments. Results shown are the means ± SE. *, *p* < 0.05 compared with the control; $, *p* < 0.05 compared with stimulants (CCK or TPA) preincubated with 1%DMSO vs. stimulants pre-incubated with IPA-3 or Pir 3,5, respectively; ∞, *p* < 0.05 compared with Pir 3,5 alone. (**F**) ROS generation was measured as previously reported [203]. The results are representative of 6 independent (*n* = 6) experiments. Results shown are the means ± SE. *, *p* < 0.05 compared with the control; N.S., not significant. These results are drawn from the figures and data in [203]. These results demonstrate that inhibition of PAK2 by the active antagonist, IPA3, but not by the inactive control Pir3,5 inhibits the experimental pancreatitis features induced by supramaximal CCK including inhibition of caspase 3,8 and 9 activation, as well as activation of trypsin and stimulation of cell necrosis and ROS generation.

Acute pancreatitis is an inflammatory disorder starting in the exocrine pancreas, which can progress to involving both peripancreatic as well as remote tissues and is one of the leading causes of gastrointestinal (GI) hospitalizations in the USA and in many countries [198,199]. Acute pancreatitis is characterized by the inflammation, edema and necrosis of pancreatic tissue, which can result in significant morbidity, as well as an overall mortality of 5% [198,199]. The results from numerous studies in different tissue demonstrate that PAKs are an important general mediator of inflammation [24,200], and thus one might suspect their activation could play an important role in acute pancreatitis, which has a prominent inflammatory component. The results from a number of recent studies prove evidence that the activation of p21-activated kinases may play important roles in the pathobiology of acute pancreatitis. In a recent study [12], using a widely used experimental model of acute pancreatitis, which is induced by supramaximal concentrations of CCK in rat pancreatic acinar cells, a number of results supported the conclusion that the activation of group I PAKs (PAK2) plays important roles in the early meditation of acute pancreatitis. The premature activation of the pancreatic digestive enzyme, trypsinogen to trypsin, in the pancreatic acinar cell is considered to be one of the main initiating events in acute pancreatitis [198,201,202]; in this experimental model of acute pancreatitis, this required the CCK activation of PAK2 (Figure 4 and Figure 7 and Table 2).

Furthermore, the activation of reactive oxygen species [ROS] has been reported to play an important role in the pathobiology of acute pancreatitis in various disease models [198,204,205], and this was also found to be dependent on the activation of PAK2 [12] (Figure 4 and Figure 7F and Table 2). In addition, in the supramaximal CCK model as well as other models of acute pancreatitis, acinar cell death can occur and is mediated by the activation of apoptosis and necrosis [198,206,207]. For the stimulation of apoptosis in acute pancreatitis, the activation of caspases 3, 8 and 9 plays an essential role, and, in this recent study [12], the activation of these caspases required the activation of PAK2 (Figure 4 and Figure 7A,C,E and Table 2). Furthermore, the activation of PAK2 was an important mediator of necrosis in this model of acute pancreatitis (Figure 4 and Figure 7B,D and Table 2) [12]. The authors of this study [12] propose that because of the multiple roles of PAK2 in the pathobiology of experimental acute pancreatitis, PAK2 could be an important therapeutic target for the treatment of acute pancreatitis.

The possible importance of group I PAKs in acute pancreatitis is further supported by a recent study in mice using an in vivo supramaximal CCK model of acute pancreatitis [67]. In this model, with the induction of acute pancreatitis, the pancreatic acinar cell group I PAKs (PAK1) were overexpressed, which led to the activation of NF-kB and the p38 signaling cascade, which in turn resulted in the pathological features of acute pancreatitis including serum concentrations of amylase and lipase and increased levels of tissue necrosis factor alpha, interleukin-6 and interleukin beta. This result is consistent with studies in fibroblasts/macrophages, wherein the activation of NF-kB has been shown to require the activation of the group I PAK, PAK1 [208]. These results led the authors to suggest that group I PAK1 inhibitors might be a potential therapy for the treatment of acute pancreatitis [67].

The possibility that the activation of group II PAKs (PAK4) could also be important in the pathogenesis of acute pancreatitis was suggested by a study [209] using the in vitro model of acute pancreatitis by administering taurolithocholic acid to the pancreatic acinar cell line, AR42J cells. This study [209] demonstrated an up-regulation of the PAK4 gene in addition to 21 other genes, raising the possibility it might be a therapeutic drug target.

The studies above providing evidence that the activation of PAKs may play an important role in acute pancreatitis are supported by studies reporting important roles for Ras, Rac and Cdc42 activation in acute pancreatitis. Rac1 and CDC42 are the main activators of PAKs (Table 1), and Ras can activate Rac/Cdc42 [210], resulting in PAK activation. These studies include results from CCK-induced acute pancreatitis [205] in mice, which demonstrated a marked amelioration of the acute pancreatitis with a Rac1 inhibitor, as well as a marked inhibition of acute pancreatitis-associated lung injury after giving the Rac1 1 inhibitor [205]. Other studies demonstrated that the inhibition of Ras signaling decreased the severity of taurocholate induced acute pancreatitis [211] and that the ameliorating effect of the flavone, baicalin, on acute pancreatitis was due in part to its inhibition of Cdc42 [212]. Because the activation of PAKs is the principal effector of the action of these small p21-GTP activators, these results also support the conclusion that the activation of PAKs is important in acute pancreatitis.

## 8. Pancreatic Tissue Growth (Exocrine, Cancer and Islets)

Previous studies have demonstrated that group I and II PAKs are involved in the signaling pathways related to growth in normal tissue [6,8,11,27,28], as well as neoplastic tissues [7,22,75,213,214]. In numerous normal tissues, several studies have shown that PAKs are involved in both physiological growth and response to injury with the activation of the MAP kinase and PI3K pathway playing prominent roles [6,8,11,27,28]. In neoplastic tissue, PAKs, particularly PAK1, PAK2 and PAK4, have a high level of expression in many cancers with PAK alterations involving gene amplification, fusions, mutations and deletions resulting in PAKs playing key roles in pancreatic cancer [2,75,76,214,215], as well as in lung, colon, gastric, prostate and breast cancer [6,22,27]. In pancreatic cancers and other tumors, PAKs have been shown to play important roles in proliferative signaling in the evasion of apoptosis and the promotion of proliferation, cancer initiation, epithelial mesenchymal transformation, cell survival, migration, invasion, metastasis, inducing angiogenesis and drug resistance and immune responses [2,22,75,215].

The key role in the stimulation of neoplastic growth and transformation in many tumors such as pancreatic cancer is the tumoral activation of Ras, which in turn activates PAKs [216,217] (Figure 4 and Figure 5 and Table 1) [6,11,22]. In pancreatic cancer, a mutation in Ras, most frequently in KRAS, is observed in >85% of pancreatic tumors, which results in a constitutively active Ras, which, in addition to activating PAKs, can activate the MAPK, PI3K, Hedgehog, Wnt and Notch signaling cascades, all of which can affect growth/proliferation [27,75,217,218,219]. KRAS, a small GTPase enzyme that functions as a signal transducer [218], participates in the downstream pathway and activation of the main growth pathway, Raf/Mek/ERK [219,220].

Similar to the well-studied roles of PAKs in the growth behavior of pancreatic cancer and other neoplasms, an important signaling role for PAKs in mediating the growth and mass of pancreatic islets has been reported [48,51,221,222]; however, the role of PAKs in exocrine pancreatic growth has not been studied until recently. Studies have shown that the release of the hormone/neurotransmitter, CCK, plays an essential role in mediating growth in pancreatic exocrine cells by interacting with the CCK_1_-R’s, resulting in the stimulation of numerous growth stimulatory signaling cascades [82,121,223,224]. Numerous studies have shown that the CCK_1_-R-mediated activation of the MAP kinase cascade plays an essential, central role in mediating the CCK stimulated growth of pancreatic exocrine cells [121,223,225,226].

**Figure 8:**
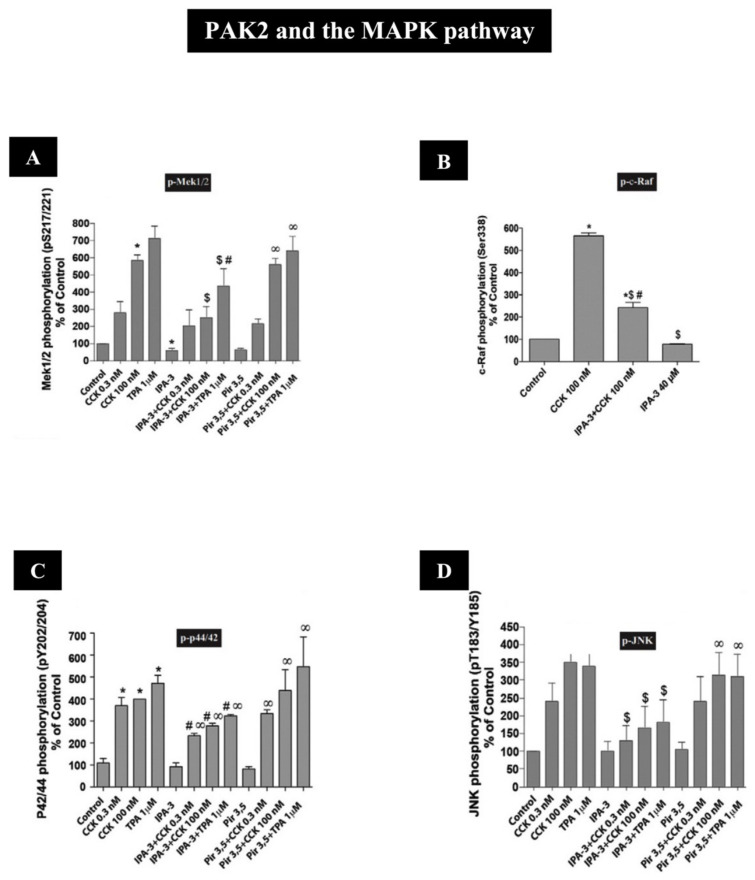
Effect of the PAK2 inhibitor, IPA-3 and its inactive analogue Pir 3,5 on the ability of physiological (0.3 nM) and supraphysiological concentrations of CCK (100 nM) and TPA to stimulate activation of Mek 1/2 (**A**), c-Raf (**B**), p42/44 (**C**) and JNK (**D**). Isolated pancreatic acini were incubated in the absence or presence of IPA-3 (40 uM) or Pir 3,5 (40 uM) for 15 min and then incubated with no addition (control) or CCK-8 (0.3 and 100 nM) for 3 min or TPA (1 uM) for 5 min and then lysed. Results are expressed as means ± SE of 4 independent experiments. Mek 1/2, c-Raf, p42/44 and JNK phosphorylation were measured by using anti-pS217/221 Mek1/2, anti-pS338-Raf, anti-pY202/204 p42/44 and anti-pT183/Y185 JNK, respectively [12]. Results are expressed as percentages of basal stimulation of the control group. *, *p* < 0.05 compared with the control; #, *p* < 0.05 compared with IPA-3 alone; ∞, *p* < 0.05 compared with Pir 3,5 alone; and $, *p* < 0.05 compared with stimulants (CCK or TPA) preincubated with 0.1% DMSO vs. stimulants pre-incubated with IPA-3 or Pir 3,5, respectively. These results are drawn from the figures and data in [12]. These results demonstrate PAK2 activation essential for CCK activation of the p42/44 MAPK, which plays a pivotal role in CCK induced acinar cell growth (adaptive growth, growth due to injury and regenerative growth after resection), as well as in CCK activation of c-Raf, Mek 1/2 and JNK.

**Figure 9:**
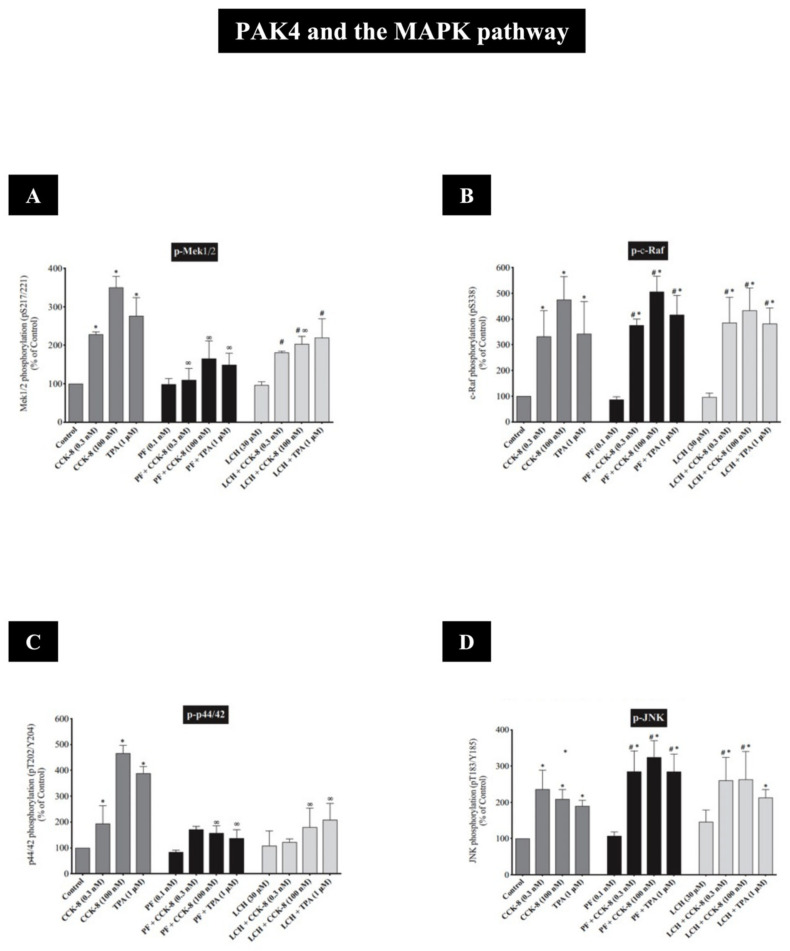
Effect of the PAK4 inhibitors, PF-3758309 and LCH-7749944 on the ability of physiological (0.3 nM) and supraphysiological concentrations of CCK (100 nM) and TPA to activate Mek 1/2 (**A**), c-Raf (**B**), p42/44 (**C**) and JNK (**D**). Isolated pancreatic acini were incubated in the absence or presence of PF-3758309 (0.1 nM) or LCH-7749944 (30 uM) for 3 h and then incubated with no addition (control) or CCK-8 (0.3 and 100 nM) for 3 min or TPA (1 uM) for 5 min and then lysed. Mek 1/2, c-Raf, p42/44 and JNK phosphorylation were measured by using anti-pS217/221 Mek1/2, anti-pS338-Raf, anti-pY202/204 p42/44 and anti-pT183/Y185 JNK, respectively [41]. Results are expressed as a percentage of basal stimulation of the control group. *, *p*< 0.05 compared with the control group; #, *p* < 0.05 compared with inhibitors alone (PF-3758309 or LCH-7749944); ∞, *p* < 0.05 compared with stimulants without inhibitors. These results are drawn from the figures and data in [41]. These results demonstrate that PAK4 activation is essential for CCK activation of the p42/44 MAPK, which plays a pivotal role in CCK induced acinar cell growth (adaptive growth, growth due to injury and regenerative growth after resection), as well as in CCK activation of Mek 1/2, but, in contrast to PAK2 activation, it does not play a role in CCK activation of c-Raf or JNK.

Recent studies in the exocrine pancreatic cells using hormonally responsive, dispersed pancreatic acinar cells demonstrate that the activation of both group I PAKs (PAK2) and group II PAKs (PAK4) in these cells is essential for mediating the activation of the ERK1/2 signaling growth cascade (Figure 4, Figure 5, Figure 8C and Figure 9C and Table 2) [12,14].

The results in these studies demonstrated that the signaling cascades for the activation of the two different groups of PAKs, in relation to the activation of the ERK1/2 cascade, differed [12,14,41] (Figure 8A and Figure 9A and Table 2). In the case of PAK2 activation by CCK_1_-R stimulation [10], the activation of c-Raf with the subsequent activation of Mek1/2 and JNK was an essential upstream effector [10] (Figure 8A,B,D and Table 2), whereas with PAK4 activation, the ERK1/2 activation is both functioning upstream as well as functioning as a downstream mediator of PAK4 activation [14,41] (Figure 5 and Figure 9C and Table 2), and Mek1/2 was also activated by PAK4 [41] (Figure 9A). However, PAK4 did not mediate c-Raf nor JNK activation [41] (Figure 9B,D). These results demonstrate that even though the two groups of PAKs have many structural similarities, in the same cell with the same stimulant (i.e., CCK), stimulating the same key signaling cascade (i.e., ERK1/2 activation), which can result in the same response (i.e., growth), the overall PAK-ERK1/2 signal cascade had some important differences.

## 9. Conclusions

The main purpose of this paper was to review recent insights into the roles of p21-activated kinases from studies of PAKs in pancreatic exocrine tissue, an area for which there is little information, except for islet function and insulin secretion.

These studies show that, in contrast to pancreatic islets and pancreatic cancer, two pancreatic tissues in which the roles of PAKs have been well studied, in the pancreatic acinar cells only one group I PAK (PAK2) and only one group II PAK (PAK4) are present. These studies show that a number of physiological stimulants, including CCK, as well as a number of growth factors, activate PAK2 and PAK4. Also, they show that both PAKs have similarities and differences in the ability of the secretagogues, including CCK, that stimulate PLC-mediated cascades and those activating the adenylate cascades (VIP/secretin) to activate PAK2 and PAK4. Furthermore, they show that the activation of both PAK2 and PAK4 by CCK is not only rapid and sustained but also required both CCK_1_ receptor states. The studies of physiological/pathological function show that PAK2 and PAK4 are involved in secretory function and activating the central growth signaling cascade involving the MAPK cascade and that PAK2 activation plays an essential role in the induction of pancreatitis. Furthermore, these studies report the signaling cascades that both activate PAKs in these cells, as well as a number of the distal post PAK signaling cascades, which in a number of cases are novel compared to what is generally found in non-secretory well-studied tissues.

One major limitation of the recent data reviewed in this paper for the possible importance of PAKs in exocrine pancreatic is that it, in large part, comes from studies in rodents. This is the case because compared to these detailed studies of the presence, signaling and function of PAKs in rat pancreatic exocrine tissue, there is, in general, only limited information on comparative studies of the presence or absence of PAKs or their roles in human pancreatic tissues or other species, except for their roles in islet/insulin secretion and pancreatic cancer. The comparison of the available data showed that, in a normal pancreas, the type of group I/group II PAK varies with different species; however, at present, the comparable roles for PAK activation and function have not been studied. This situation is not unique to these studies because, in the case of the cell biology of exocrine pancreatic function, both normally and in diseases such as pancreatitis, almost all the information has come from studies on rats and mice, and, when compared to limited human data, the rodent data have generally provided very important insights, which are also seen with some variation in human tissues. Although no data exist in PAKs for exocrine pancreatic tissues for such a comparison at present, both the above results in other pancreatic exocrine cell biology studies and the limited information from studies of PAKs in human islet/insulin secretion compared to that from rodent islets support the general conclusion that although there may be some variation, the insights from the rodent data are generally applicable to the human situation, except the cell pathway, which may show variations. Furthermore, except for islet cell function, there are no data in the literature from any study that clearly address the point of species differences on PAK function in a given organ. The only data we could find are from studies of PAKs’ importance in insulin secretion from different species. These studies show that in rat, mouse and human islets, reported in the current paper, there can be different PAKs present. However, the studies show that in the different species, PAK1, PAK2 or PAK4 can regulate insulin secretion, suggesting that different group I or group II PAKs may regulate similar islet functions in different species, but at least one is important in their regulation in each species studied.

In conclusion, this paper reviews information from recent studies that show that both group I and group II PAKs could play an important role in the exocrine pancreas by participating in secretory and growth cascades. These results combined with a few isolated reports of the roles of PAKs in other exocrine/secretory tissues suggest that both groups of PAKs could play a much larger role in exocrine/secretory tissues than generally thought at present and thus should be studied in this context in the future. Furthermore, because PAK2 and PAK4 have been related to pancreatic cancer, these two p21-activated kinases could be considered a prognostic and immunotherapeutic marker pancreatic cancer; therefore, future research in this direction should be necessary.

## Figures and Tables

**Table 1:** Characteristics of group I and II p21-activated kinases.

	Group I PAKs	Group II PAKs	References
Members	PAK1, PAK2 and PAK3	PAK4, PAK5 and PAK6	[1,2,3]
Function	Cell survival, apoptosis, cell motility, protein synthesis, glucose homeostasis, secretion, growth and cellular proliferation	Regulation of cell morphology, cytoskeletal organization, cell proliferation, cell cycle control, migration, secretion, growth and survival	[4,6,10,11,12,13,14,15,16,17,20]
Activators	Cdc42/Rac	Cdc42 > Rac	[1,2,6,8,27,28]
Phosphorylation site	Thr423 in PAK1 Thr402 in PAK2 Thr421 in PAK3	Ser474 in PAK4 Ser602 in PAK5 Ser560 in PAK6	[1,2,8,9,10,27,28,29,30]
Methods of activation	The autoinhibitory domain (AID) overlaps with the PBD, and together they act as a dimer. Active Cdc42 or Rac binds to the PBD, disrupting the interaction between the AID and the PBD, leading to a conformational change of PAK becoming a monomer, which subsequently becomes autophosphorylated	Model I: Active Cdc42 binds to the PBD and causes a conformational change; PAK4 exists as a monomer in the inactive state and remains inactive due to the binding of the kinase domain and the AID-like sequence. Model II: Reduction in PSD autoinhibition mediated by SH3 proteins; Cdc42 binds to the PBD, reorienting it and allowing the PSD to bind to SH3 proteins, resulting in the reduction in autoinhibition and kinase activation.	[1,2,8,10,20,27,28,29,31,32]

**Table 2:** Regulation and interactions of PAK2 and PAK4 in rat pancreatic acini.

	PAK2	PAK4	References
Most used inhibitors	IPA-3 (allosteric) FRAX597 (ATP competitive)	PF-3758309 (ATP competitive) LCH-7749944 (ATP competitive)	[33,34,35,36,37,38,39]
Stimulation by pancreatic secretagogues	CCK-8, carbachol, bombesin	CCK-8, carbachol, bombesin, endothelin-1, VIP, secretin	[10,14,15]
Stimulation by pancreatic growth factors	EGF, bFGF, PDGF	EGF, IGF, HGF, EGF, bFGF, PDGF	[10,14]
Stimulation by post-receptor activators	TPA	TPA; thapsigargin, A23187, 8-Br-cAMP, forskolin	[10,14,15]
CCK1 receptor state: EC_50_ of CCK-8 (nM) EC_50_ of CCK-JMV (nM)	0.44 ± 0.05 0.18 ± 0.14	0.052 ± 0.003 0.10 ± 0.01	[10,14]
Signaling pathways	PYK2, p125^FAK^; paxillin, p130^CAS^; c-Raf, Mek1/2, p44/42, JNK; p85PI3K, Akt (reversed inhibition), p70S6K (reversed inhibition); caspases 3, 8, 9; trypsin activity; LDH release; ROS generation	PYK2, p125^FAK^; paxillin, p130^CAS^; Mek1/2, p44/42, GSK3, ß-catenin; PP2A, cofilin	[10,12,14,40,41]
Exocrine function	Growth; amylase release	Growth; amylase release; fluid secretion (Na^+^, K^+^-ATPase)	[10,14,15]

**Table 3:** Similarities and differences in the ability of pancreatic secretagogues, growth factors and post-receptor activators to activate group I or group II PAKs in different tissues compared to pancreatic acinar cells.

Group I PAKs	Group II PAKs	References
Similarities with pancreatic acinar cells		[10,14,15]
Pancreatic hormones/secretagogues/neurotransmitters		
Carbachol stimulated PAK1 in Cos7 cells	No data	[92]
Endothelin did not activate group I PAKs in myocytes	No data	[93]
No data	Activation of some GPCRs, such as those for ß-adrenergic agents, prostaglandins and α-MSH, can stimulate PAK4 activation via cAMP in HEK293 cells, B16 melanoma cells and MCF7	[94,95,96]
No data	Different hormones can activate PAK4, such as thyroid-stimulating hormones in papillary thyroid cancer; C-X-C motif chemokine 12 in prostate cancer; and α-MSH in B16 melanoma cells	[96,97,98]
Pancreatic growth factors		
Insulin did not stimulate PAK1/PAK2 in NIH-3T3 cells	Insulin activates PAK4 in epithelial cells	[99,100,101,102]
No data	IGF-1 can active PAK4	[9,29]
No data	HGF activates PAK4 in epithelial cells	[100,101,102]
EGF activated PAK2 in mouse skin epidermal cells and PAK1 in Cos7 cells	EGF activates PAK4 in epithelial cells	[92,100,101,102,103]
bFGF stimulated PAK1/PAK2 phosphorylation in NIH-3T3 cells and stimulated cell growth by activating PAK1/PAK2 in PC-12 cells	No data	[99,104,105,106]
PDGF stimulated PAK1/PAK2 phosphorylation in NIH-3T3 cells	PDGF can active PAK4	[9,29,99]
Group I PAKs are activated in a PLC-dependent manner by angiotensin II in vascular smooth muscle cells, gastrin in colorectal cancer cells and colorectal mucosa cells	No data	[107,108]
Group I PAKs are activated in a PLC-dependent manner by gastrin in colorectal cancer cells and colorectal mucosa cells		[109,110]
Group I PAKs are activated by Rac1, which is a PAK activator in other tissues, as well as group I PAKs’ activation by muscarinic cholinergic agents in fibroblasts or neuroblastoma cells and in smooth muscle cells	No data	[16,92,111,112]
Post-receptor activators		
No data	8-Br-cAMP activated Cdc42 in human mesangial cells, which is the principal upstream activator of PAK4	[1,2,6,8,27,28,113]
No data	Forskolin can activate PAK4 in papillary thyroid cells and in prostate cancer cells	[97,114]
Differences with pancreatic acinar cells		
Pancreatic hormones/secretagogues/neurotransmitters		
Not data	Not data	
Pancreatic growth factors		
Group I PAKs mediate IGF-1 and insulin signaling in mesothelial cells and in mouse endocrine L cells	No data	[115,116]
HGF regulated PAK1/PAK2 in prostate cancer and epithelial cells	No data	[117]
Post-receptor activators		
No data	No data	

Abbreviations: Cos7, monkey kidney fibroblast-like cell line; GPCR, G-protein-coupled receptor; HEK293, human embryonic kidney 293 cell line; B16, melanoma cell line; MCF7, breast cancer cell line; 8-Br-cAMP, 8-Bromoadenosine 3′,5′-cyclic monophosphate sodium salt.

## Data Availability

No new data have been reported or analyzed in this study.

## Abbreviations

AC, adenylyl cyclase; AID, autoinhibitory domain; Akt, protein kinase B; AP-1, activator protein-1; ATP, adenosine triphosphate; bFGF, basic fibroblast growth factor; Ca^2+^, calcium; cAMP, cyclic adenosine monophosphate; CCK, COOH-terminal octapeptide of cholecystokinin; CCK_1_-R, CCK 1 receptor; Cdc42, cell division control protein 42; DAG, diacylglycerol; EGF, epidermal growth factor

EPAC, exchange protein activated by cyclic AMP; FAK, focal adhesion kinase.

FRAX597, small-molecule pyridopyrimidinone-group-1-PAK-inhibitor; GF, growth factor; GI, gastrointestinal; GSK3, glycogen synthase kinase 3; GTPase, guanosine-triphosphate-binding protein; HGF, hepatocyte growth factor; IGF, insulin-like growth factor; IPA-3, 1,1′-dithiodi-2-naphthtol; JNK, c-Jun-N-terminal kinase; LCH, LCH-7749944; LDH, lactate dehydrogenase; MAPK/ERK/p44/42, mitogen-activated protein kinase; MARCKS, myristoylated alanine-rich C kinase substrate; MCP-1, monocyte chemotactic protein-1; Mek, MAP kinase; MIP-1α, macrophage inflammatory protein-1α; NF-kB, nuclear factor kB; PAK, p21-activated kinases; PBD, p21-binding domain; PDGF, platelet-derived growth factor; PF, PF-3758309; PI3K, phosphatidylinositol 3-kinase; PKA, protein kinase A; PKC, protein kinase C; PKD, protein kinase D; PLC, phospholipase C; p38, p38 mitogen-activated protein kinase; p70S6K, p70 ribosomal S6 kinase; p125^FAK^, focal adhesion kinase p125(Fak); p130^CAS^, adaptor protein p130; ROS, reactive oxygen species; Pi, inorganic phosphatase; PI3K, phosphoinositide 3-kinase; PP, protein phosphatase; PSD, pseudo-substrate domain; PTEN, phosphatase and tensin homolog; PYK2, proline-rich tyrosine kinase 2; Raf, proto-oncogene serine/threonine-protein kinase proto-oncogene; RANTES, regulated on activation, normal T cell expressed and secreted; ROS, reactive oxygen species; Ser and S, serine; SFK, Src family of kinases; SHC, Src homology 1 domain containing transforming protein; SH3, SRC homology 3; STAT3, signal transducer and activator of transcription-3; Thr and T, threonine; TPA, 12-O-tetradecanoylphorbol-13-acetate; VIP, vasoactive intestinal polypeptide; 8-Br-cAMP, 8-Bromoadenosine 3′,5′-cyclic monophosphate sodium salt; 8-CPT-2-Me-cAMP, 8-(4-chlorophenylthio)-2’-O-methyladenosine 3′,5′-cyclic monophosphate sodium.
